# Pyroelectric Nanogenerator Based on an SbSI–TiO_2_ Nanocomposite

**DOI:** 10.3390/s22010069

**Published:** 2021-12-23

**Authors:** Krystian Mistewicz

**Affiliations:** Institute of Physics—Center for Science and Education, Silesian University of Technology, Krasińskiego 8, 40-019 Katowice, Poland; krystian.mistewicz@polsl.pl; Tel.: +48-32-603-41-56

**Keywords:** antimony sulfoiodide, titanium dioxide, nanowires, pyroelectric effect, nanogenerator, renewable energy

## Abstract

For the first time, a composite of ferroelectric antimony sulfoiodide (SbSI) nanowires and non-ferroelectric titanium dioxide (TiO_2_) nanoparticles was applied as a pyroelectric nanogenerator. SbSI nanowires were fabricated under ultrasonic treatment. Sonochemical synthesis was performed in the presence of TiO_2_ nanoparticles. The mean lateral dimension *d_a_* = 68(2) nm and the length *L_a_* = 2.52(7) µm of the SbSI nanowires were determined. TiO_2_ nanoparticles served as binders in the synthesized nanocomposite, which allowed for the preparation of dense films via the simple drop-casting method. The SbSI–TiO_2_ nanocomposite film was sandwiched between gold and indium tin oxide (ITO) electrodes. The Curie temperature of *T_C_* = 294(2) K was evaluated and confirmed to be consistent with the data reported in the literature for ferroelectric SbSI. The SbSI–TiO_2_ device was subjected to periodic thermal fluctuations. The measured pyroelectric signals were highly correlated with the temperature change waveforms. The magnitude of the pyroelectric current was found to be a linear function of the temperature change rate. The high value of the pyroelectric coefficient *p* = 264(7) nC/(cm^2^·K) was determined for the SbSI–TiO_2_ nanocomposite. When the rate of temperature change was equal *dT*/*dt* = 62.5 mK/s, the maximum and average surface power densities of the SbSI–TiO_2_ nanogenerator reached 8.39(2) and 2.57(2) µW/m^2^, respectively.

## 1. Introduction

Efficiency in thermal energy harvesting is an important challenge to produce green energy for sustainable development. Most of the waste heat generated in industry is available at low temperatures, generally below 373–503 K [1]. Low-temperature waste heat is especially difficult to recover successfully using currently available technologies [2]. Thermal energy can be converted into electric energy by applying electrochemical systems [3], thermogalvanic cells [4,5], thermoelectric [6,7,8], thermomagnetic [9,10], and pyroelectric generators [11,12,13]. However, all mentioned technologies suffer from low efficiency and limited reliability. Therefore, new materials and engineering concepts must be proposed and developed in the field of low-temperature waste heat recovery to ensure their future large-scale and commercial application.

Inorganic ferroelectric crystals or ceramics [14,15], ferroelectric polymers [16], and non-ferroelectric compounds [17] are three main groups of materials commonly used to construct pyroelectric generators. Material morphology is a key factor affecting the pyroelectric performance of the device. The efficiency of thermal energy conversion to electrical power can be enhanced through quantum confinement in pyroelectric nanomaterials [18]. Thus, there is growing interest in the development and examination of pyroelectric nanogenerators based on thin films [19], nanowires [20,21], or nanoparticles [22].

Antimony sulfoiodide (SbSI) is a ternary compound that possesses ferroelectric properties with a Curie temperature of 295 K [23]. The first report on the pyroelectric properties of SbSI single crystals was provided by Imai et al. [24]. The large pyroelectric coefficient (1.2 µC/(cm^2^·K)) of bulk single crystals of SbSI was measured over a temperature range encompassing the ferroelectric phase transition [25]. The significantly much lower pyroelectric coefficients of 8.06 pC/(cm^2^·K) and 180 nC/(cm^2^·K) were determined for SbSI films grown by flash evaporation [26] and physical vapor deposition [27], respectively. However, the pyroelectric properties of SbSI nanowires have not been investigated so far. Recently, nanowires of SbSI and other compounds that belong to the chalcohalide family of materials have received a great amount of attention due to the fact of their possible applications in piezoelectric nanogenerators for mechanical energy harvesting [28,29], underwater ultrasounds sensors [30], photovoltaic devices [31,32], piezo- and photocatalysis [33,34].

Many different techniques have been proposed till now to fabricate antimony sulfoiodide films including electron beam evaporation [35], flash evaporation [36,37], physical vapor deposition [27], pulsed laser deposition [38,39], molecular beam epitaxy [40], solution processing [41], and combined solution and vapor processes [42,43]. The majority of the aforementioned techniques possess serious drawbacks such as complexity or the need to use high-temperature treatments. A typical way to fabricate continuous films of SbSI nanowires is based on the application of a binding polymer matrix, e.g., polyacrylonitrile (PAN) [32]. As a result, the electrical properties of such a prepared composite can be worse in comparison to the electrical properties of pristine SbSI nanowires. Ye and co-workers [44] presented sol-gel fabrication of thin films and bulk solids of SbSI micro-crystallite-doped organically modified TiO_2_. The optical absorption spectrum of this material exhibited an evident quantum confinement effect [44], whereas the electrical properties of the material were not examined. It should be noted that the application of TiO_2_ nanoparticles in composites for pyroelectric nanogenerators has been reported in the literature as a factor improving pyroelectric performance [45].

In this paper, a facile fabrication of the SbSI–TiO_2_ nanocomposite in one step is presented for the first time. TiO_2_ nanoparticles were used as binders in the synthesized nanocomposite. The dense film of the SbSI–TiO_2_ nanocomposite was prepared using a simple drop-casting method and sandwiched between the electrodes. Such a device was applied as a pyroelectric nanogenerator.

## 2. Materials and Methods

### 2.1. Material Synthesis and Nanogenerator Fabrication

**Figure 1:**
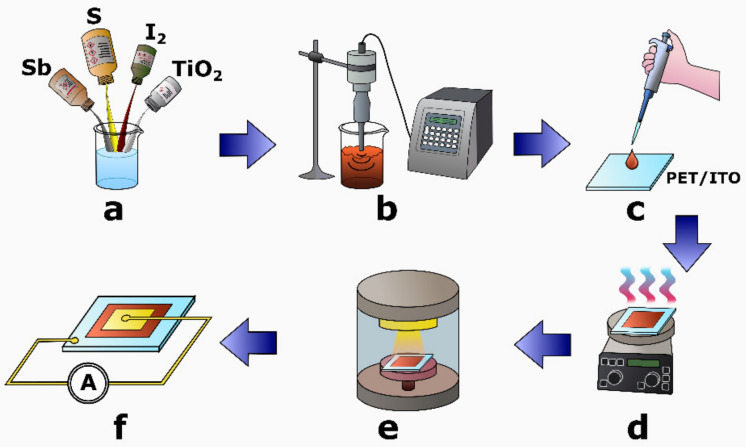
Schematic fabrication process flow of the SbSI–TiO_2_ pyroelectric nanogenerator. A detailed description is given in the text.

Figure 1 presents the preparation of the pyroelectric nanogenerator. In the first stage, the chemical elements (i.e., antimony, sulfur, and iodine) were weighted in a stoichiometric ratio. The reagents (i.e., 868 mg of Sb, 227 mg of S, and 921 mg of I_2_) were mixed with titanium dioxide (TiO_2_) nanoparticles purchased from Merck KGaA. According to the data provided by the material manufacturer, the average size of the TiO_2_ nanoparticles was 21 nm. The mass of TiO_2_ (860 mg) was adjusted to constitute approximately 30% of the total mass of the nanocomposite. The mixture was added to the beaker containing 10 mL of pure ethanol (Figure 1a). Such a prepared suspension of the reagents was subjected to ultrasonic irradiation, generated by the VCX-750 ultrasonic reactor (Sonics & Materials, Inc., Newtown, CT, USA) as shown in Figure 1b. The material synthesis was carried out at a temperature of 323 K over 4 h. After the synthesis was completed, the orange gel of the SbSI–TiO_2_ nanocomposite was drop-casted onto an indium tin oxide (ITO) coated polyethylene terephthalate (PET) substrate as depicted in the Figure 1c. The drop-casting deposition of the material was performed in several steps using an electronic pipette. The sample was then placed on a hot plate and dried at a temperature of 333 K (Figure 1d). When the sample drying was finished, the 200 nm thick gold electrode was sputtered on the top side of the SbSI–TiO_2_ nanocomposite (Figure 1e) by applying a Q150R ES rotary pumped coater (Quorum Technologies Ltd., Laughton, United Kingdom). Finally, the prepared SbSI–TiO_2_ nanogenerator was connected to the external measurement circuit (Figure 1f).

### 2.2. Structural and Chemical Characterization of the SbSI–TiO_2_ Nanocomposite

The morphology and chemical composition of the SbSI–TiO_2_ nanocomposite were investigated using scanning electron microscopy (SEM) and energy-dispersive X-ray spectroscopy (EDS), respectively. The experiments were performed using a Phenom Pro X microscope (Thermo Fisher Scientific, Waltham, MA, USA) integrated with an EDS spectrometer. The SEM studies were carried out at the acceleration voltage of 10 kV, whereas the EDS survey was accomplished at 15 kV. The EDS data were analyzed using the ProSuite Element Identification computer program (Thermo Fisher Scientific).

### 2.3. Electrical Measurements

The SbSI–TiO_2_ nanocomposite was inserted into the SH-242 environmental test chamber (Espec) to examine its electrical properties. All experiments were carried out at a constant relative humidity (*RH*) of 50%. The sample was tested under dark conditions to eliminate the influence of excess carries photogeneration on the measured electric signals. The change in temperature was achieved via air flow inside the chamber. The measurements were performed at a standard atmospheric pressure (1 atm). It was confirmed for the SbSI nanowires that their electrical properties are sensitive to the various gases including oxygen and hydrogen [46]. However, the air circulation inside the chamber was closed. Thus, the gas composition of the air remained the same. As a result, the effect of the gas on the electrical properties of the sample was eliminated. Before pyroelectric investigations, the SbSI–TiO_2_ nanocomposite was poled to align the electric dipoles in the SbSI nanowires and enhance the pyroelectric output of the nanogenerator. It was conducted by cooling the device below the Curie temperature and applying an external electric field of 12 kV/m, which is higher than the coercive field of SbSI [23]. The pyroelectric response of the SbSI–TiO_2_ nanocomposite was registered for different rates of temperature change (d*T*/d*t*) in the range from 1.8 to 62.5 mK/s. The low values of d*T*/d*t* were chosen to avoid possible interference caused by the thermoelectric effect [47]. The electric output of the SbSI–TiO_2_ nanocomposite was measured with the 6430 Sub-Femtoamp Remote SourceMeter (Tektronix, Beaverton, OR, USA). Data acquisition was performed using a PC computer with a GPIB bus and an appropriate program in LabView (National Instruments, Austin, TX, USA).

## 3. Results and Discussion

**Figure 2:**
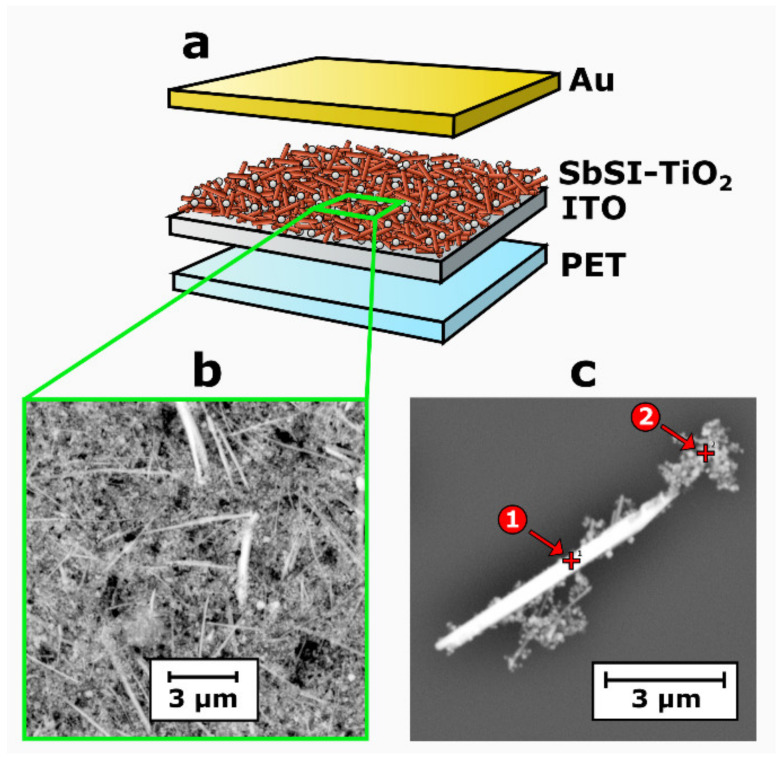
The layer structure of the pyroelectric nanogenerator (**a**), SEM micrograph of the SbSI–TiO_2_ film (**b**), and a selected nanowire (**c**).

Figure 2a presents the prepared pyroelectric nanogenerator based on the SbSI–TiO_2_ nanocomposite sandwiched between the Au electrode and ITO-coated PET. A typical SEM micrograph of the SbSI–TiO_2_ nanocomposite is shown in Figure 1b. This material consisted of randomly distributed one-dimensional nanocrystals of SbSI and agglomerations of TiO_2_ nanoparticles. The concentrations of the chemical elements were averaged over the sample. The EDS survey proved that titanium dioxide constituted 29.9% of the total mass of the nanocomposite, which is in good agreement with the initial amount of TiO_2_ (30%) used for material preparation. TiO_2_ nanoparticles served as fillers in the synthesized nanocomposite, which allowed for preparing dense films via a facile drop-casting method. Many attempts have been made to fabricate films containing only SbSI nanowires. However, they were not successful. The presence of voids in the layers of pure SbSI nanowires (without TiO_2_ nanoparticles) resulted in the formation of the short-circuited samples after sputtering of the gold electrodes. Another way to fabricate a continuous film of SbSI nanowires is by applying a polymer as a binder [32]. Such a method possesses a significant drawback. It was found in [32] that the increase in polymer concentration in the SbSI–PAN nanocomposite led to an undesirable reduction in its electrical conductivity and photovoltaic performance.

The elemental atomic ratio of 0.37:0.36:0.27 for Sb, S, and I averaged over nanocomposite volume was close to the stoichiometric composition of SbSI (Table 1). The theoretical values should be 0.33 for each of the elements in this ternary compound. No other elements were detected in the sample. The small amount of material was deposited on the Si substrate. An individual SbSI nanowire was separated from the nanocomposite to examine its chemical composition (Figure 2c). The EDS signal for Si was subtracted from the atomic concentrations evaluated for other components. The elemental atomic ratio registered for spot 1 in Figure 2c corresponded to the chemical composition expected for antimony sulfoiodide (Table 1). The values of atomic concentrations determined for spot 2 in Figure 2c indicate the presence of agglomerated TiO_2_ nanoparticles and slight traces of antimony. It should be noted that an excess amount of Sb is frequently reported in the literature for nanowires [32,34], nanocrystals [33], and thin films [27] of SbSI. This effect can be attributed to the fact that the surface of crystalline SbSI nanowires is surrounded by fuzzy shells, which have a chemical composition that may be different from the concentrations of elements in the core of the nanowire [32]. Atomic concentrations of titanium and oxygen are in agreement with the theoretical values for stoichiometric TiO_2_ (Table 1). Since titanium dioxide exhibits excellent chemical stability [48,49], it should be resistant to ultrasonic treatment.

**Figure 3:**
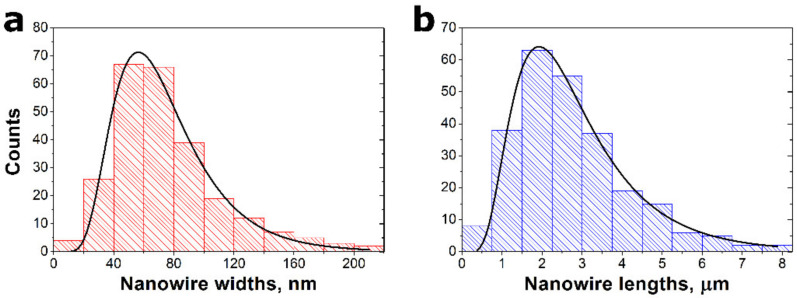
Distributions widths (**a**) and lengths (**b**) of SbSI nanowires in the SbSI–TiO_2_ nanocomposite. The black lines represent log-normal functions expected for the nanoscale object size distribution described by Equation (1). The values of the fitted parameters of Equation (1) are given in the text.

The prepared material was characterized with scanning electron microscopy to determine the average diameter and length of the SbSI nanowires as presented in Figure 3. Manual image analysis was performed on 250 randomly selected nanowires. The diameters and lengths vary in the wide ranges, reaching up to 220 nm and 8.2 µm, respectively. One can see that the distribution of nanowires sizes followed a log-normal function [50,51]:

(1) $$f\left(x\right)=\frac{A}{\sqrt{2\pi \cdot }\sigma \cdot x}\exp\left[-\frac{{\left[\ln\left(\frac{x}{x_{a}}\right)\right]}^{2}}{2\sigma^{2}}\right],$$

where *A* is a constant, *x* means the size of the nanowires (diameter or length), *x_a_* denotes the average value of the nanowire size, and *σ* is its standard deviation. It should be underlined that the log-normal function is frequently reported in the literature as a relation describing size distributions of nanowires [50,51,52] and nanoparticles [53,54,55]. The mean diameter *d_a_* = 68(2) nm and the length *L_a_* = 2.52(7) µm of the SbSI nanowires were determined. The obtained value of d_a_ is equal within the measurement uncertainty to the average diameter (69(3) nm) of the SbSI nanowires reported in [32].

**Figure 4:**
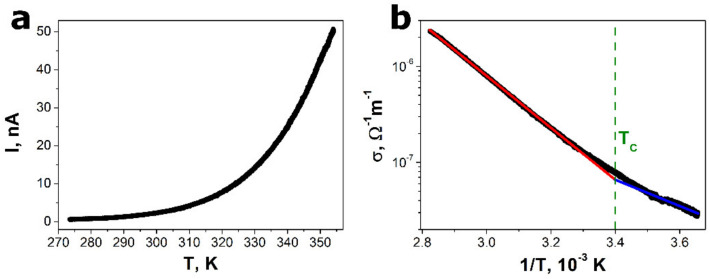
The temperature dependence of the electric current registered under a constant bias voltage of 0.1 V (**a**) and the Arrhenius plot electrical conductance of the SbSI–TiO_2_ nanocomposite (**b**). The solid red and blue lines in (**b**) represent the best fitted theoretical dependence (2) in the paraelectric and ferroelectric phases, respectively. The green, dashed line indicates the reciprocal value of the determined Curie temperature. The values of the fitted parameters of Equation (2) are given in the text.

The influence of temperature on the electric current flowing through the SbSI–TiO_2_ nanocomposite under constant bias voltage is shown in Figure 4a. The rise in temperature leads to a sharp enhancement of the electric current. It is a typical behavior for a semiconducting material. Moreover, when the temperature increases, the reduction in the grain boundary resistance can result in the reduction in the barrier for the mobility of charge carriers participating in grain boundary conduction [56]. Taking into account the geometrical dimensions of the investigated sample, the electric conductance of the SbSI–TiO_2_ nanocomposite was calculated (Figure 4b). It is well known that in the case of ferroelectric material, the Arrhenius plot of an electric conductance consists of two linear curves with different slopes corresponding to the paraelectric and ferroelectric phases [56,57]. Thus, a change in activation energy is observed near Curie temperature (*T*_C_) [58,59,60]. This effect was also documented in the literature for thin films of SbSI [35,61]. Two different theoretical curves were least squares fitted to the experimental results, shown in Figure 4b, using a well-known relation [56,57]:

(2) $$\sigma \left(T\right)=\sigma_{0}\cdot \exp\left(-\frac{E_{A}}{k_{B}T}\right),$$

where *σ*_0_ is the pre-exponential coefficient, *E_A_* means an activation energy, and *k*_B_ denotes the Boltzmann constant. The values of activation energies of *E_A_* = 0.537(1) eV and *E_A_* = 0.271(2) eV were determined for the paraelectric and ferroelectric phases, respectively. They could not be compared with the literature data for SbSI due to the fact that these parameters are thickness dependent [61]. Finding the intersection of the linear dependencies in the Arrhenius plot of an electric conductance (Figure 4b) allowed for the estimation of the Curie temperature of the examined material (*T_C_* = 294(2) K). The obtained value was consistent with the data from the literature for ferroelectric SbSI listed in Table 2.

**Figure 5:**
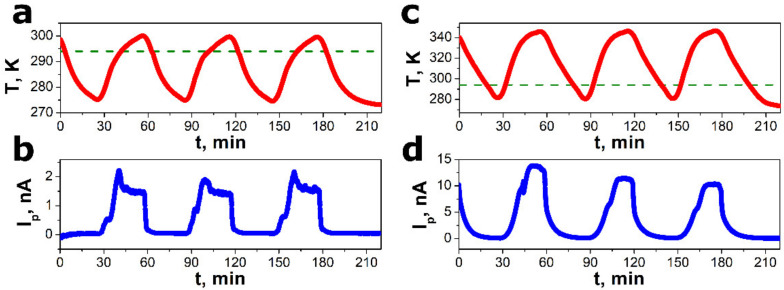
Cyclic changes in temperature (**a**,**c**) and corresponding output current (**b**,**d**) of the SbSI–TiO_2_ pyroelectric nanogenerator. The green horizontal line represents the value of the Curie temperature *T_C_* = 294(2) K.

The sample of the SbSI–TiO_2_ nanocomposite was subjected to periodic temperature fluctuations as presented in Figure 5a,c. The experiments were performed for different amplitudes of temperature changes. A rectangular-shaped transient characteristic of pyroelectric current was observed (Figure 5b,d) when the triangular temperature waveform was applied to the SbSI–TiO_2_ nanogenerator. Such behavior is well documented in the literature for other pyroelectric materials, e.g., PVDF–TiO_2_ composite [45], PVDF–ZnO nanocomposite [63], Bi_0.5_Na_0.5_TiO_3_–P(VDF–TrFE) nanocomposite [64], P(VDF–TrFE) [65], PbTiO_3_–P(VDF–TrFE) [66], LiNbO_3_–polypropylene [67], CeO_2_-doped Na_0.5_Bi_0.5_TiO_3_ ceramics [68], BaZr_0.2_Ti_0.8_O_3_/Ba_0.7_Ca_0.3_TiO_3_ heterostructures [69], and CdS nanorods [47]. This proves that the measured electric response of the SbSI–TiO_2_ nanocomposite was due to the true pyroelectric effect. It should be noted that only SbSI nanowires contributed to the pyroelectric response of the nanocomposite. The non-ferroelectric inclusion of TiO_2_ cannot be polarized, since its net dipole moment is almost zero [45]. A slight decrease in the amplitude of the pyroelectric response, shown in Figure 5d, can be attributed to the so-called “aging effect” [70].

The only positive pyroelectric response of the SbSI–TiO_2_ nanogenerator is observed (Figure 5b,d) due to the fact that the rectifying *p*–*n* heterojunction was formed between the SbSI nanowires and the ITO electrode as well between the SbSI nanowires and the TiO_2_ nanoparticles. Usually, SbSI is regarded as *p*-type semiconductor [31,37,61,71,72], whereas both ITO [73] and TiO_2_ [74] exhibit *n*-type electrical conductivity. The work functions of ITO, TiO_2_, and SbSI were 4.4–4.5 eV [75], 4.2–4.7 eV [76], and 5 eV [31,71,72], respectively. Accordingly, since the work function of *p*-type SbSI is higher than that of n-type ITO and *n*-type TiO_2_, a built-in electric field was formed in the ITO/SbSI and TiO_2_/SbSI interfaces. When the temperature increased (*dT*/*dt* > 0), a positive pyroelectric potential was formed. Thus, the ITO/SbSI and TiO_2_/SbSI interfaces were forward-biased pn junctions. In such a case, the electrons could flow freely across the interfaces, and the pyroelectric current was measured in the external circuit. When the device cooled down (*dT*/*dt* < 0), the ITO/SbSI and TiO_2_/SbSI interfaces were reversely biased barriers, leading to almost zero electric output of the nanogenerator. This is the process of creating, separating, preserving, and accumulating charges [77]. As a result, the device generated a rectified electric output. The same effect is observed in the case of direct-current generators based on *p*–*n* [78,79,80,81,82] or Schottky [77,83,84,85] junctions. Morozovska et al. [20] proposed that the rectification effect of the junction barrier allows the application of a ferroelectric nanowire array fixed between flat electrodes as the direct current generator. Moreover, one should remember that the built-in electrical field of a *p*–*n* junction barrier can create a polar axis in the solid [86]. The pyroelectric effect can be enhanced, as the electric dipole moment, due to the charge separation at the junction, depending on the temperature mainly through the temperature dependence of the dielectric constant [86].

**Figure 6:**
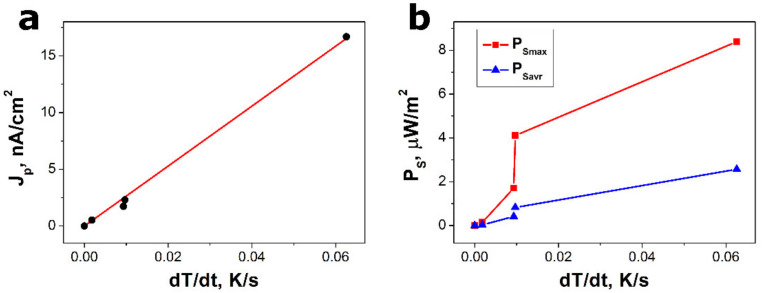
The output pyroelectric current density (**a**) and the surface power density (**b**) of the SbSI–TiO_2_ pyroelectric nanogenerator as a function of the temperature change rate. The red line in Figure (**a**) represents the best fit dependence (3). The red and blue experimental points in Figure (**b**) show the values of the maximum and average surface power density, respectively.

The magnitude of the pyroelectric current depends on the rate of temperature change (*dT*/*dt*). It is described by the following equation [11,12,13]:

(3) $$I_{p}=\frac{dQ}{dt}=pA\frac{dT}{dt},$$

where *Q* represents a pyroelectric charge, *p* denotes the pyroelectric coefficient, and *A* means the electrode area. The pyroelectric current density (*J_p_* = *I_p_*/*A*) is presented as a function of the rate of temperature change in Figure 6a. One can see that the experimental results followed a linear dependence. According to Equation (3), the slope of *J_p_* versus *dT*/*dt* was equal to the pyroelectric coefficient. The value of the pyroelectric coefficient of *p* = 264(7) nC/(cm^2^·K) was determined for the SbSI–TiO_2_ nanocomposite by fitting the theoretical Formula (3) to the experimental data in the graph in Figure 6a.

The maximum power density (*P_S_*_max_) of the SbSI–TiO_2_ nanogenerator was calculated using the peak values of the output pyroelectric current (*I_p_*_max_), voltage (*U*_max_), and electrode area of the device (*A*):

(4) $$P_{S\max}=\frac{U_{\max}\cdot I_{p\;\max}}{A}.$$

One can see that the maximum surface power density is proportional to the maximum value of the pyroelectric current density. The average values of the surface power density (*P_S_*_avr_) were determined using the following equation:

(5) $$P_{S\mathrm{avr}}=\frac{P_{\mathrm{avr}}}{A}=\frac{1}{A\cdot \Delta t}\cdot \int_{0}^{\Delta t}U\cdot I_{p}dt,$$

where Δ*t* represents the period time of a heating–cooling cycle. Figure 6b shows that the increase in the temperature change rate resulted in the obvious rise of *P_S_*_max_ and *P_S_*_avr_. The pyroelectric coefficient, the maximum and average power densities, calculated for the SbSI–TiO_2_ nanocomposite, were compared with the values of these parameters reported in the literature for other pyroelectric materials (Table 3). They were divided into five main groups, i.e., non-ferroelectric materials, inorganic ferroelectric bulk crystals or ceramics, pure ferroelectric polymers, ferroelectric thin films or nanomaterials, and ferroelectric composites. It should be underlined that the SbSI–TiO_2_ nanocomposite had the highest values for the pyroelectric coefficient and surface power density among all the ferroelectric composites as listed in Table 3. Furthermore, the pyroelectric performance of the SbSI–TiO_2_ nanocomposite was much better than that achieved for the thin films of SbSI [26,27] and nanowires of antimony selenoiodide (SbSeI) [87], which is an isostructural material for SbSI [88]. It is expected that the incomplete coverage of the TiO_2_ on the SbSI nanorods should lead to the formation of pores between these two different materials. A matrix–void composite with low permittivity is desired for pyroelectric applications, exhibiting a high figure of merit [89,90]. Therefore, the high pyroelectric performance of the SbSI–TiO_2_ nanocomposite can be attributed to the good pyroelectric properties of SbSI as well as to the incomplete coverage of the TiO_2_ on the SbSI nanorods.

Electrical energy (Δ*E_V_*) generated by a unit of the volume (*V*) of the SbSI–TiO_2_ nanocomposite during the time interval (*t*) was evaluated applying a formula given below:

(6) $$\Delta E_{V}=\frac{1}{V}\cdot \int_{0}^{t}U\cdot I_{p}dt.$$

**Figure 7:**
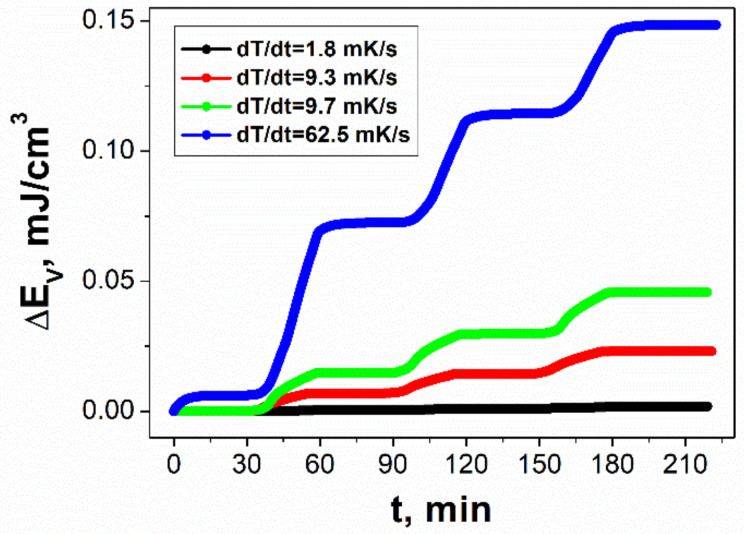
Volume energy density generated by the SbSI–TiO_2_ device during three periodic thermal input cycles measured for different rates of temperature change (● 1.8 mK/s; ● 9.3 mK/s; ● 9.7 mK/s; ● 62.5 mK/s).

Figure 7 presents a time dependence of the volume energy density generated by the SbSI–TiO_2_ device during three periodic thermal input cycles. An increase in the rate of temperature change leads to an enhancement of the Δ*E_V_*. When the temperature difference (Δ*T*) was equal to 25 K, the electrical energy produced per unit volume of the SbSI–TiO_2_ nanocomposite per cycle amounted to 15.2 µJ/cm^3^. This value was lower than Δ*E_V_* = 230 µJ/cm^3^ achieved for the PVDF film per single thermal cycle at the temperature change Δ*T* = 23 K [93].

The probable impact of the size of the TiO_2_ nanoparticles on the pyroelectric efficiency of the SbSI–TiO_2_ nanocomposite can be explained as follows. A decrease in a TiO_2_ nanoparticle’s size leads to a reduction in the electrical conductivity of this nanomaterial [106]. Wei and co-workers [107] presented that lower conductivity of the ceramic inclusion in the ferroelectric composite results in higher polarization, which is favorable to the poling of the composite. Therefore, the enhancement of the pyroelectric performance of the SbSI–TiO_2_ nanocomposite should be observed with the decrease of the TiO_2_ nanoparticle’s size.

## 4. Conclusions

For the first time, a method of fabrication of the SbSI–TiO_2_ pyroelectric nanogenerator was described. It involved the sonochemical preparation of the SbSI–TiO_2_ nanocomposite in a single step and its drop-casting deposition on ITO-coated PET. TiO_2_ nanoparticles served as fillers in the synthesized nanocomposite, which were crucial to obtain dense films with the desired morphology. It is a new alternative approach to fabricate devices based on nanowires. This method eliminates the need for application of a polymer matrix as a binder. The proposed technology is simple and universal. It can be used for preparation of nanogenerators from other one-dimensional chalcohalide nanostructures.

The SEM and EDS studies of the SbSI–TiO_2_ nanocomposite confirmed the formation of stoichiometric SbSI nanowires with an average diameter and length of 68(2) nm and 2.52(7) µm, respectively. The Curie temperature of 294(2) K was determined for the examined material, which is in good agreement with the data reported in the literature for ferroelectric bulk crystals, thin films, and pristine nanowires of SbSI.

The SbSI–TiO_2_ nanocomposite sandwiched between the ITO and Au electrodes was subjected to periodic temperature fluctuations. The registered pyroelectric response was well correlated to the temperature changes. The measured signal originated from the pyroelectric properties of SbSI nanowires. Non-ferroelectric TiO_2_ nanoparticles were unable to contribute to the electric output of the nanogenerator, since they cannot be polarized. The pyroelectric current density was found to be a linear function of the rate of temperature change. The large pyroelectric coefficient of 264(7) nC/(cm^2^·K) was determined for the SbSI–TiO_2_ nanocomposite. This value was higher than the pyroelectric coefficient reported in the literature for many different pyroelectric materials including ZnO nanowires, PZT bulk ceramics, BaTiO_3_ bulk ceramics, LiNbO_3_ single crystal, PVDF polymers, SbSI thin films, and nanowires of SbSeI, which is an isostructural material to the SbSI. The maximum and average surface power densities of the SbSI–TiO_2_ nanogenerator reached 8.39(2) and 2.57(2) µW/m^2^, respectively. The electrical energy produced per unit volume of 15.2 µJ/cm^3^ was obtained per one thermal cycle for which the temperature change was equal to 25 K. It is expected that the values of the aforementioned parameters can be enhanced by adjusting the weight concentration of the titanium dioxide in the nanocomposite. Such investigations will be performed in the near future. The results, presented in this paper, demonstrate that the ferroelectric SbSI–TiO_2_ nanocomposite has great potential for use in pyroelectric sensors and thermal energy harvesters.

## Figures and Tables

**Table 1:** The chemical composition determined for the areas of the sample shown in Figure 2b,c.

Chemical Element	Atomic Concentration, %		
	Area in Figure 2b	Spot 1 in Figure 2c	Spot 2 in Figure 2c
Sb	15.6	36.8	13.8
S	14.9	30.4	0
I	11.1	32.8	0
Ti	18.5	0	28.8
O	39.9	0	57.4

**Table 2:** A comparison of the determined Curie temperature of the SbSI–TiO_2_ nanocomposite with literature data for SbSI.

Material	Preparation Method	*T_C_*, K	Reference
bulk crystal of SbSI		295	[23]
bulk crystal of SbSI	vapor phase growth	292–293	[24]
bulk crystal of SbSI	Bridgman method and vapor transport technique	293	[25]
SbSI film	physical vapor deposition	295	[27]
SbSI film	electron beam evaporation	294	[35]
SbSI film	flash evaporation	293	[37]
SbSI film	pulsed laser deposition	292	[38]
SbSI film	pulsed laser deposition	290–294	[39]
SbSI nanowires	sonochemical synthesis	291(2)	[62]
SbSI–TiO_2_ nanocomposite	sonochemical synthesis	294(2)	this paper

**Table 3:** Comparison of the pyroelectric performance achieved for the SbSI–TiO_2_ nanocomposite and other materials (BCs—bulk ceramics; BNT—Bi_0.5_Na_0.5_TiO_3_; NPs—nanoparticles; NRs—nanorods; NWs—nanowires; *p*—pyroelectric coefficient; P_S_—surface power density; PVC—poly(vinyl chloride); PVDF polyvinylidene difluoride; P(VDF–TrFE)—poly(vinylidenefluoride-co-trifluoroethylene); PZT—lead zirconate titanate; SC—single crystal; TFs—thin films). The abbreviations “max” and “avr” refer to the maximum and average surface power densities, respectively.

Group of Materials	Material	*p*, nC/(cm^2^·K)	*P_S_*, µW/m^2^	Reference
non-ferroelectric materials	ZnO NWs	1.5		[91]
	ZnO TFs	1.0–1.4		[92]
	CdS NRs	470		[47]
inorganic ferroelectric bulk crystals or ceramics	PZT BCs	53.3	3700 ^max^	[93]
	PZT BCs	20	13.6 ^avr^	[94]
	BaTiO_3_ BCs	10	2240 ^max^	[95]
	BaTiO_3_ BCs	16		[96]
	LiNbO_3_ SC	5–8	219 ^max^	[97]
	SbSI SC	1200		[25]
pure ferroelectric polymers	PVDF	1.94		[45]
	PVDF	4	108 ^max^	[98]
	PVDF		0.13 ^max^	[99]
	P(VDF–TrFE)	2.4		[65]
	P(VDF–TrFE)	4.39	128 ^max^	[100]
ferroelectric thin films or nanomaterials	Ba_0.8_Sr_0.2_TiO_3_ TFs	25		[101]
	KNbO_3_ NWs	0.8		[102]
	SbSI TFs	0.008		[26]
	SbSI TFs	180		[27]
	SbSeI NWs	44(5)	0.59(4) ^max^	[87]
ferroelectric composites	BaTiO_3_–PVC	10.6		[103]
	PVDF–diamond NPs	8.7		[104]
	PVDF–TiO_2_	2.45		[45]
	PVDF–ZnO NPs	~2.9		[63]
	PVDF–CH_3_NH_3_PbI_3_	0.004	1.75 ^max^	[105]
	P(VDF–TrFE)–BNT NPs	5		[64]
	P(VDF–TrFE)–PbTiO_3_ NPs	4		[66]
	SbSI NWs–TiO_2_ NPs	264(7)	8.39(2) ^max^ 2.57(2) ^avr^	this paper
